# LSVT-BIG therapy in Parkinson’s disease: physiological evidence for proprioceptive recalibration

**DOI:** 10.1186/s12883-020-01858-2

**Published:** 2020-07-11

**Authors:** Manuel Peterka, Thorsten Odorfer, Michael Schwab, Jens Volkmann, Daniel Zeller

**Affiliations:** 1 Department of Neurology, University of Würzburg, Josef-Schneider-Str. 11, 97080 Würzburg, Germany; 2 Stiftung Bürgerspital zum Hl. Geist, 97070 Würzburg, Germany

**Keywords:** Proprioception, Amplitude, Training, Pointing error, LSVT-big therapy

## Abstract

**Background:**

There is growing evidence for proprioceptive dysfunction in patients with Parkinson’s disease (PD). The Lee Silvermann Voice Treatment-BIG therapy (LSVT-BIG), a special training program aiming at an increase of movement amplitudes in persons with PD (PwPD), has shown to be effective on motor symptoms. LSVT-BIG is conceptionally based on improving bradykinesia, in particular the decrement of repetitive movements, by proprioceptive recalibration.

**Objective:**

To assess proprioceptive impairment in PwPD as compared to matched controls and to probe potential recalibration effects of the LSVT-BIG therapy on proprioception.

**Methods:**

Proprioceptive performance and fine motor skills were assessed in 30 PwPD and 15 matched controls. Measurements with significant impairment in PwPD were chosen as outcome parameters for a standardized 4 weeks amplitude-based training intervention (LSVT-BIG) in 11 PwPD. Proprioceptive performance served as primary outcome measure. Secondary outcome measures included the motor part of the MDS-UPDRS, the nine-hole-peg test, and a questionnaire on quality of life. Post-interventional assessments were conducted at weeks 4 and 8.

**Results:**

Compared to the control group, PwPD showed significantly larger pointing errors. After 4 weeks of LSVT-BIG therapy and even more so after an additional 4 weeks of continued training, proprioceptive performance improved significantly. In addition, quality of life improved as indicated by a questionnaire.

**Conclusion:**

LSVT-BIG training may achieve a recalibration of proprioceptive processing in PwPD. Our data indicates a probable physiological mechanism of a symptom-specific, amplitude-based behavioral intervention in PwPD.

## Background

Parkinson’s Disease (PD) is one of the most common neurodegenerative diseases [1]. Bradykinesia, defined as a slowness of movement and a progressive reduction of frequency or amplitude of repetitive movements, is the key symptom of PD [2]. Clinical manifestations of this “poverty of movement” [3] are gait impairment with reduced arm swing and smaller steps, difficulties to rise from a chair, loss of spontaneous movements like gesturing, and hypomimia [4, 5]. Bradykinesia has been shown to be the clinical feature to correlate best with the degree of dopamine deficiency [6].

Impairment of a variety of neuronal pathways has been brought forward to explain the pathophysiology behind bradykinesia and its link to dopamine depletion. One of the main features associated with bradykinesia is the altered processing of sensory input [7–9]. In particular, proprioceptive input is needed for the accuracy of amplitude and speed of executed movements and has been shown to be altered in PD [10–12]. Pathological processing of proprioceptive information may thus be a key pathological mechanism of bradykinesia [13–15].

Physiotherapy is commonly recommended for people with PD (PwPD), aiming at a reduction of PD symptoms or even at a modification of disease outcome [16], although overall evidence for an effectiveness of standard physiotherapy in PD is rather weak [16, 17]. This may be mainly attributed to the high variability, but low specificity of the training protocols. To head for a standardized, symptom-specific training for people with PD, Farley and Koshland published the protocol of an amplitude-based behavioral intervention (called “LSVT-BIG” therapy) improving speed-amplitude scaling relations across the upper and lower limbs (Farley & Koshland, 2005). Notably, in a randomized, controlled trial, the Lee Silvermann Voice Treatment-BIG therapy (LSVT-BIG) effected significant improvements of motor functioning in people with PD, thereby proving superior to other training protocols (Ebersbach et al., 2010). The primary objective of the LSVT-BIG training is to overcome small movement amplitudes in order to normalize motor function [18]. While this is initially achieved by repetitive explicit (conscious) modulation of movement amplitudes, the transfer to a more implicit pattern of large movements has been postulated to rely on proprioceptive recalibration. However, this mode of action has not been inquired and therefore remains speculative.

Here, we aimed to identify suitable measures of proprioceptive impairment in PwPD as compared to matched healthy controls, in order to suitably test the hypothesis that the repetitive amplitude training of LSVT-BIG acts by recalibrating pathological processing of proprioceptive input in PD.

## Methods

The protocol conformed to the principles of the declaration of Helsinki and was approved by the Ethics Committee of the Medical Faculty at the University of Würzburg with the reference number 299/14. Written informed consent was obtained from all patients.

### Participants

Thirty PwPD, according to the Movement Disorder Society diagnostic criteria [2], were recruited from the Department of Neurology, University of Würzburg. Neurological or psychiatric conditions other than PD led to exclusion from the study. All PwPD received dopaminergic treatment. In addition, a control group of 15 age-matched healthy volunteers was recruited.

Handedness was assessed by the Oldfield Handedness Inventory [19]. The Parkinson Neuropsychometric Dementia Assessment (PANDA) [20] was used to screen for cognitive impairment and depression. Frontal dysexecutive syndrome was ruled out by the German version of the Frontal Assessment Battery (FAB) [21]. All participants gave their written informed consent for participation in the study. Demographic and clinical data are shown in Table 1.

**Table 1:** Demographic and clinical data of people with Parkinson’s disease and controls

	All PwPD (*n* = 30)	BIG (*n* = 11)	Controls (*n* = 15)
Age (years)	64.8 ± 7.3	69.8 ± 6.1	65.0 ± 5.6
Sex (female/male)	10/20	3/8	10/5
LED (mg)	614 ± 394	518 ± 295 $	N/A
FD (years)	4.6 ± 3.9	3.6 ± 2.5	N/A
UPDRS III (at baseline) ^a^	18 [12 to 24]	23 [12 to 25]	N/A
Handedness (right/ ambidextrous/left)	26/4/0	8/3/0	15/0/0
PANDA (normal/MCI/ dementia likely)	24/2/4	8/1/2	14/1/0
FAB (frontal impair-ment: yes/no)	0/30	0/11	0/15

*PwPD* people with Parkinson’s disease, *LED* Levodopa Equivalent Dose ($ no significant LED change over the time period of investigation), *FD* first diagnosis, *UPDRS III* motor subscale of the MDS Unified Parkinson’s Disease Rating Scale, *PANDA* Parkinson Neuropsychometric Dementia Assessment, *MCI* mild cognitive impairment, *FAB* Frontal Assessment Battery, *N/A* not applicable

^a^ = given as median (range)

### Primary outcome measure: proprioceptive performance

Proprioceptive performance was assessed with a goniometer, a modified version of the “Wrist Position Sense Test” which has successfully been used to measure proprioceptive performance in stroke patients [22].

The goniometer was made out of two half-moon shaped wooden boards, put one over another stabilized by acrylic glass, thus creating a space in between in which a mobile cast with a handlebar could be moved radially. An indicator sliding along a slit in the acrylic glass pointed at the current position of the cast on a scale at the outside and allowed passive movements of the cast by the examiner (Suppl. Fig. 1).

The subject was seated in front of the goniometer. The upper limb (clinically more affected side) was placed upon the cast while visual control of the limb was prevented by a cloth. Three different types of examinations were conducted: two pointing tasks (LED, ARROW) and one position estimation task (PASSIVE). (i) LED: The subject was asked to point at one of six LED lights placed on the upper edge of the goniometer. Each assessment consisted of 18 pointing movements (3 times per position). (ii) ARROW: One of three round-shaped arrows (15°, 30°, 45°) was displayed on a 23-in. wide computer screen. The subject was asked to transfer the length of the arrow into a movement on the Goniometer. Each arrow was shown three times. (iii) PASSIVE: The subject’s upper limb was moved passively by the examiner via the indicator to one out of five possible positions (5°, 20°,30°,40°, and 50°), with each position presented twice. The subject was asked to indicate the arm position on a scale placed on the edge of the goniometer. The scale was only used for the PASSIVE task.

The accuracy of the pointing tasks was measured by the difference of the correct position of the LED light or length of the computer-displayed arrow and the position indicated by the subject (pointing error). Accordingly, the accuracy of the PASSIVE test was measured by the difference of the position of the upper limb estimated by the subject and the correct position (estimation error). All upper limb movements were inward rotations at the shoulder joint, as considered to be the physiological movement with the broadest achievable angular movement.

Pointing tasks were additionally carried out under dual task condition, with an acoustic task consisting of counting either high-pitched (554 Hz) or low-pitched (220 Hz) tones played randomly on common computer loudspeakers at a mean frequency of 15 tones per minute (OpenSesame 2.9.6, Portland, Oregon).

### Secondary outcome measures: fine motor skills, MDS-UPDRS III, PDQ-39

In order to assess transferability of proprioceptive training, three tasks of fine motor skills were carried out: (i) Nine-hole-peg test (NHPT) [23]: The average time of two turns was taken. (ii) Spiral drawing on a computer tablet (SPIRAL test): Participants were asked to trace a spiral on a computer tablet, using the freeware *Neuroglyphics* (neuroglyphics.org). The average time of two turns was taken [time]. In addition, as a measure of accuracy of spiral drawing, “First Order Smoothness” [FOS] [24] was calculated using Matlab (The MathWorks, Inc.,Natick, Massachusetts, USA). (iii) Writing of “elel” on a computer tablet (ELEL test): Participants were asked to copy two phrases of “elel” (each consisting of 13 ‘e’s and 13 ‘l’s) from a sample to a computer tablet, also using the freeware *Neuroglyphics*. Writing speed [speed] was calculated and also the amplitude [amplitude] and width [width] of each letter “l” was taken and summed up as a measurement for dysgraphia [25] using Matlab.

Motor impairment was quantified by means of the Unified Parkinson’s Disease Rating Scale, part III (MDS-UPDRS III) [26]. Assessments were carried out by a rater blinded for stage and type of intervention.

Quality of life was assessed by the PDQ-39 questionnaire which contains 39 questions concerning mobility, daily life activities, emotional well-being, stigma, social support, cognitive functions, communication, and dysesthesia of the body [27].

### LSVT-BIG training

Eleven of the thirty recruited PwPD started a LSVT-BIG therapy. Participation was based on recommendation of LSVT-BIG by their neurologist, willingness to participate, and availability of transport to the therapy venue. The therapy consisted of 16 one-hour training units, four of them each week. One training unit was divided in half an hour of standardized whole-body movements with maximal amplitude, repetitive multidirectional movements, and stretching. The second half of exercise includes goal-directed activities of daily living according to individual needs and preferences [28]. After intervention, all participants were asked to keep up a LSVT-BIG training routine for 30 min daily and keep a training diary.

### Experimental procedure

**Fig. 1:**
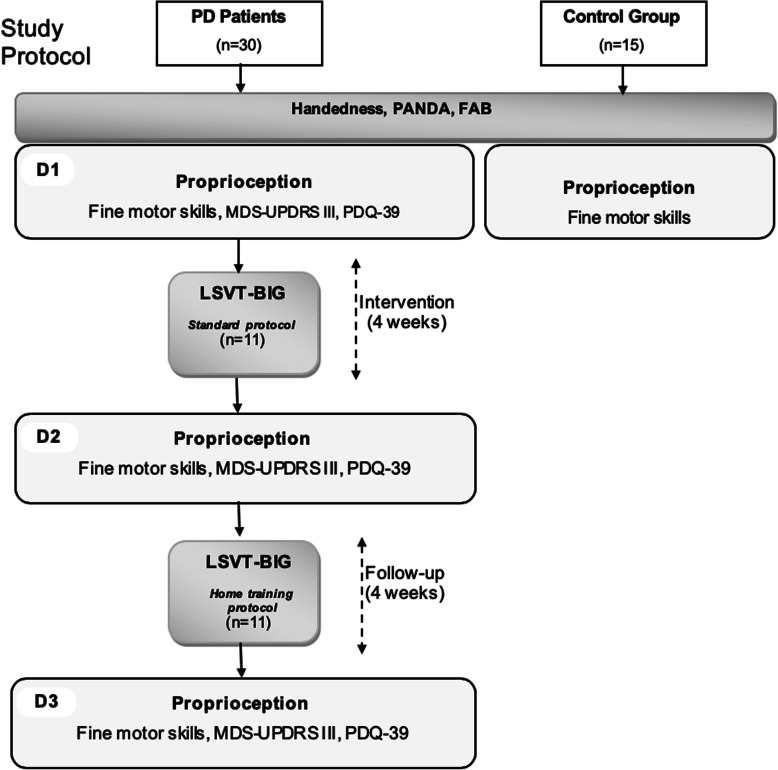
Study protocol. Flowchart of primary and secondary outcome measurements for PwPD vs. controls and for BIG on D2 and D3

A flowchart of the study protocol is shown in Fig. 1. Thirty PwPD and 15 controls were included in our study. Eleven PwPD participated in the BIG intervention. At baseline (D1), after initial assessment (handedness, PANDA and FAB), proprioception tasks were carried out. Furthermore, fine motor skills, MDS-UPDRS III, and PDQ-39 were assessed as secondary outcome measurements. Follow up was conducted at 4 weeks (D2, end of intervention) and 8 weeks (D3).

### Data analysis

We used IBM SPSS software for statistical analyses. Depending whether normal distribution was accepted (Shapiro Wilk test), t-tests or Mann-Whitney-U tests were applied for comparisons between PwPD and controls. Bonferroni-Holm correction was used to account for multiple comparisons between PwPD and controls.

Repeated-measures analyses of variance (ANOVA) were used for statistical analysis of the BIG group, and t-tests were applied for post-hoc analysis.

If not stated otherwise, data are given as mean ± SD. Effects were considered significant if *p* < 0.05.

## Results

### PwPD vs. controls: proprioceptive performance & fine motor skills

The results of the comparison between PwPD and controls are shown in Table 2. PwPD showed significantly greater pointing errors than controls in both pointing tasks (LED and ARROW). This remained unchanged by the dual task condition. Performance of PwPD and controls in the PASSIVE task was comparable between groups.

**Table 2:** Proprioceptive performance and fine motor skills: comparison between people with Parkinson’s disease and controls

		PwPD	*Controls*	*p-value*
LED test [°]	w/o dt	4.3 ± 2.1	2.4 ± 0.8	**< 0.001**^**a**^
	with dt	4.7 ± 2.2	3.0 ± 1.6	**0.006**^**a**^
ARROW test [°]	w/o dt	12.7 ± 3.5	6.5 ± 2.4	**< 0.001**^**a**^
	with dt	12.5 ± 4.4	5.9 ± 3.3	**< 0.001**^**a**^
NHPT [s]		25.4 ± 4.0	20.1 ± 2.7	**< 0.001**^**a**^
SPIRAL test, time [s]		38.2 ± 15.9	28.1 ± 6.2	0.011
SPIRAL test, FOS		1.40 ± 1.0	1.15 ± 0.4	0.754
ELEL test, speed [mm/s]		8.2 ± 4.7	9.5 ± 3.2	0.107
ELEL test, amplitude [cm]		1.23 ± 0.5	1.34 ± 0.4	0.458
ELEL test, width [cm]		1.07 ± 0.6	1.35 ± 0.4	0.011

*PwPD* Persons with Parkinson’s disease, *dt* dual task condition, *NHPT* nine-hole-peg test, *FOS* First Order Smoothness

^a^ significant after Bonferroni-Holm correction

Regarding the fine motor skill tasks, PwPD performed worse in the NHPT compared to controls. There was no significant difference between PwPD and controls in the SPIRAL-test [Time], the SPIRAL test [FOS], the ELEL test [speed], the ELEL-test [width], and the ELEL test [amplitude].

### LSVT-BIG: impact on proprioceptive performance

According to their training diaries, all patients kept up a LSVT-BIG training routine during the follow-up period, with approximately 30 min daily practice on at least 5 days a week.

Only those measurements with significant differences between PwPD and controls were chosen as outcome parameters for the BIG intervention.

**Fig. 2:**
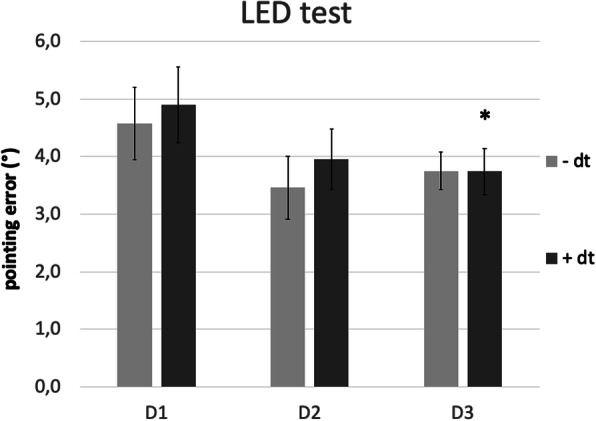
Proprioceptive performance of the BIG group at baseline, on D2 and D3. LED test, without dual task (−dt) and with dual task (+dt). Error bars indicate standard error. *p < 0.05 for post-hoc t-tests in case ANOVA indicated significant improvement

Repeated-measures ANOVA for data from D1, D2, and D3 showed a significant improvement in the LED test under dual task conditions (F(2;20) = 4.1; *p* = 0.033), but not for the LED test without dual task (F(2;20) = 2.1; *p* = 0.149). Post-hoc analysis for LED test with dual task showed a significant difference at D3 (− 1.2° [4.9° to 3.7°], *p* = 0.040, but not at D2 (− 0.9° [4.9° to 4.0°], *p* = 0.061; Fig. 2).

**Fig. 3:**
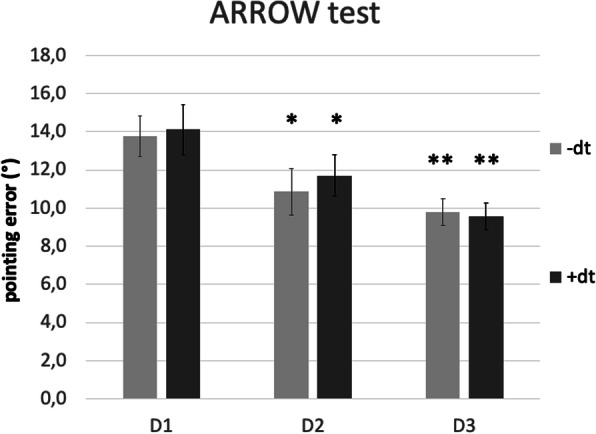
Proprioceptive performance of the BIG group at baseline, on D2 and D3. ARROW test, without dual task (−dt) and with dual task (+dt). Error bars indicate standard error. *p < 0.05 and **p < 0.01 for post-hoc t-tests in case ANOVA indicated significant improvement

In the ARROW test, repeated measures ANOVA indicated significant improvements of pointing errors both without (F(2;20) = 7.2; *p* = 0.004) and with (F(2;20) = 12.7; *p* < 0.001) dual task. Post-hoc analysis for data without dt showed a significant improvement on D2 (− 2.9° [13.8° to 10.9°], *p* = 0.026) and D3 (− 4.0° [13.8° to 9.8°], *p* = 0.002). Post-hoc analysis for data under dual task conditions yielded similar results (D2: − 2.4° [14.1° to 11.7°], *p* = 0.022; D3: − 4.6° [14.1° to 9.6°], *p* = 0.001; Fig. 3).

There was no significant correlation between changes of proprioceptive performance and changes of the MDS-UPDRS motor score.

### LSVT-BIG: impact on secondary outcome measures

Results of the secondary outcome measures are summarized in Table 3.

**Table 3:** Secondary outcome measures in the BIG group: comparison to baseline

Measure (ANOVA_RM_)	*D1*	*D2 §*	*D3 §*
MDS-UPDRS III (F(2;20) = 3.2, p = 0.061)	22.3 ± 12.3	18.3 ± 9.3	17.8 ± 10.5
PDQ-39 (F(2;20) = 4.6, *p* = 0.043*)	19.3 ± 10.3	13.8 ± 6.5 (*p* = 0.016*)	12.1 ± 8.6 (*p* = 0.052)
NHPT [s] (F(2;20) = 3.2, p = 0.063)	25.3 ± 3.3	23.5 ± 4.3	23.5 ± 2.6

*MDS-UPDRS III* motor subscale of the MDS Unified Parkinson’s Disease Rating Scale, *PDQ* Parkinson’s Disease Questionnaire, *NHPT* nine-hole-peg test, *ANOVA*_*RM*_ = *p*-values for repeated measures ANOVA

§ *p*-values for post-hoc analysis (compared to baseline), only given in case of significant ANOVA result

Repeated measures ANOVA for data from D1, D2, and D3 showed a statistically significant difference for the PDQ-39 quality of life questionnaire (F(2;20) = 4.6; *p* = 0.043). Post-Hoc analysis indicated a significant improvement at D2 (*p* = 0.016) and a strong trend towards an improvement at D3 (*p* = 0.052).

While there were trends towards improvements, repeated measures ANOVA failed to show a statistically significant improvement for the MDS-UPDRS III (F(2;20) = 3.2; *p* = 0.061), as well as for the NHPT (F(2;20) = 3.2; *p* = 0.063).

## Discussion

The present study aimed to shed light on the physiological mechanisms of LSVT-BIG therapy, a repetitive amplitude training for PwPD. In particular, we hypothesized that LSVT-BIG may act by recalibrating pathological processing of proprioceptive input. To this end, we identified two measures of proprioceptive performance which showed clear impairment in PwPD as compared to matched healthy controls. After a 4 weeks protocol of LSVT-BIG therapy and even more so after an additional 4 weeks of continued training, performance in these pointing tasks improved significantly. In addition, there was a significant improvement of quality of life as indicated by the PDQ-39 questionnaire.

Various earlier studies have investigated proprioception in PD. Overall, it has been demonstrated that PwPD perform pointing tasks less accurately than healthy controls [29–31]. It is believed that proprioceptive impairment in PD is due to altered processing of proprioceptive information on a subcortical and/or cortical level [32], whereas muscle spindles show normal morphology in PD [32]. Clinical and neuroanatomical studies conclude that sensorimotor integration in the basal ganglia is altered in PwPD [9, 13, 15, 33]. More specifically, an impairment of proprioceptive-motor integration in the basal ganglia is discussed [15].

Our finding of poor performance in the pointing tasks is in line with these results and concurs with the theory of altered integration of proprioceptive information in PD.

In contrast, we did not find differences between PwPD and controls in the PASSIVE task. This task is restricted to the sensory perception of proprioceptive information, while the pointing tasks comprise an additional motor component (active movement) and sensorimotor integration. Previous studies proposed impaired perception of proprioceptive information to be an independent symptom of PD occurring at early stages [10–12], possibly due to an altered connection between somatosensory cortex and basal ganglia [32]. However, comparison between studies is limited by methodological differences which may refer to the question at which joint proprioception is assessed [12] and whether static (*where does the hand point to?*) or dynamic (*which is the smallest angle of constant passive movement to be perceived?*) aspects of position sense are tested [10, 11]. While we cannot completely rule out interference factors such as muscle activation during the measurements, as we did not employ EMG control, we are confident that thorough instruction and behavioural monitoring assured proper performance.

With respect to fine motor skills, PwPD performed significantly worse in the NHPT as compared to controls. This is well in line with impaired dexterity due to PD [34]. In contrast, despite a general trend towards lower amplitudes and speed in PwPD, group differences in the tablet-based tests of drawing and writing did not survive correction for multiple comparisons. Most likely, the subdivision of those tests into partial aspects of drawing and writing, like speed, width, or smoothness, entailed a loss of sensitivity for overall motor differences, which were better caught by the NHPT.

Altogether, our first step findings support the theory of altered sensorimotor integration and confirm impaired dexterity in PwPD.

In a second step we addressed the question of whether LSVT-BIG therapy has an impact on those measures of proprioception which had turned out to be imparied in PwPD. More specifically, we hypothesized that the repetitive amplitude training of LSVT-BIG acts by recalibrating proprioceptive impairment in PD. Indeed, as to both active pointing tasks, we found a reduction of pointing errors after LSVT-BIG treatment. In other words, PwPD who had engaged in repetitive amplitude training were able to move their arm more precisely. This was particularly evident by significant improvements in the ARROW test, irrespecitve of whether performed with or without dual task. In the LED test, where subjects were asked to point to a visible goal, differences were only significant under dual task conditions, with a strong trend for D2 and a significant improvement for D3. Importantly, both tests were performed without visual feedback of the actual arm position. Thus, multisensory integration was required to match LED position or arrow length (both vision) and arm position (proprioception), respectively. However, while the LED test happened at the same level within a reaching circuit, ARROW required the abstract process of translating the length of an arrow into a proportional radial arm movement. This implies two variables which might respond to repetitive amplitude training: the command preceding the movement, and perception of the proprioceptive feedback generated by the movement.

To further discriminate, pointing tasks were also assessed under dual task conditions. Dual tasking is expected to increase error rates when performance relies on conscious effort, whereas the error rate should remain stable if performance is mainly based on implicit learning [35–37]. Of note, performance under dual task conditions was virtually identical to performance without dual task. This may reinforce the notion that LSVT-BIG training works by modulating pathological movement patterns on a subcortical level, leading to a long-term improvement of proprioceptive skills in PwPD.

While the particular mechanisms underlying proprioceptive improvement are beyond the scope of this study, we may speculate on where neural plasticity occurs: On the one hand, the integration of somatosensory input might be optimized on the basal ganglia level by reorganizing dopaminergic pathways which have been shown to be involved in sensorimotor impairment in PwPD [9, 33]. This is supported by PET data during Lee Silvermann Voice treatment (LSVT), the logopedic analog to LSVT-BIG [38]. On the other hand, training might modulate the perception of somatosensory input on the cortical level in order to overcome impaired sensorimotor integration in the basal ganglia. Similar mechanisms of learning have been shown in patients after limb amputation [39] or stroke [40].

It might seem surprising that improvements in the proprioceptive tasks were even more obvious at D3, i.e. following an additional 4 weeks during which patients kept up amplitude-based training without further guidance. However, this observation indicates that continuous practice of the BIG principles may implicate their consolidation, thereby facilitating their implicit application. Accordingly, Ebersbach et al. suggested a dose dependency of whether LSVT-BIG training would obtain a movement recalibration or not [41]. Importantly, as no visual or verbal feedback was provided to the patients during proprioceptive assessments, improvements over time cannot be simply explained by learning effects due to repetition.

At the level of secondary outcome measures, changes of the MDS-UPDRS III did not reach significance. While earlier studies have already shown efficacy of the LSVT-BIG training on this clinical scale [18, 28, 41, 42], our study was not powered to replicate those findings. Nevertheless, there was a strong trend towards an improvement whose size (between 4 and 5 points) appeared comparable. Whether the lack of correlation between improvements of proprioceptive performance and MDS-UPDRS III can be attributed to only partially overlapping neural underpinnings or to insufficient power remains an open question.

Similarly, performance in the NHPT did not change significantly in our group of PwPD while a previous prospective, uncontrolled study had brought evidence for an improvement of gross manual dexterity following LSVT BIG [41]. It seems remarkable, however, that this effect has been described at week 16 after treatment initiation. It therefore cannot be ruled out that the transfer of the amplitude-based training towards distal, i.e. fine motor movements, would require more than 8 weeks of training and follow-up.

Overall, amplitude-based training was associated with a significant improvement of quality of life. As this effect outlasted the 4 weeks of guided therapy, we interpret it to be a treatment effect rather than an unspecific effect of the higher level of social interaction [43].

## Conclusions

In conclusion, our findings concur with the theory of altered sensorimotor integration in PwPD. Moreover, they lend support to the hypothesis that LSVT-BIG retunes proprioceptive processing. Confirmation in cohorts with higher numbers of participants in a controlled design, preferably by means of a cross-over study, is needed. Nevertheless, to our knowledge, this is the first study to indicate a probable physiological mechanism of a symptom-specific, amplitude-based behavioral intervention in PwPD. Above LSVT-BIG, it eventually may encourage further studies investigating the impact of other treatment protocols on proprioceptive performance in PwPD.

## Supplementary information

**Additional file 1: Supplemental Figure.** Schematic view of the goniometer. Proprioceptive tasks were carried out in a sitting position with a cloak impeding visual feedback. Upper limb position was indicated by a scale at the back of the goniometer. For clarity reasons, the handlebar within the mobile cast is not shown.

## Data Availability

The datasets used and/or analyzed during the current study are available from the corresponding author on reasonable request.

## Abbreviations

PD: Parkinson’s disease
PwPD: Persons with Parkinson’s disease
BIG: LSVT-BIG therapy group
PANDA: Parkinson Neuropsychometric Dementia Assessment
FAB: Frontal Assessment Battery
NHPT: Nine-hole-peg test
MDS-UPDRS III: Unified Parkinson’s Disease Rating Scale, part III
