# Supplementary material for: LSVT-BIG therapy in Parkinson’s disease: physiological evidence for proprioceptive recalibration
Source: BMC Neurol. 2020 Jul 11;20:276. doi: 10.1186/s12883-020-01858-2 (PMC7353788; doi:10.1186/s12883-020-01858-2)
Supplement: Supplementary file 1 — Additional file 1: Supplemental Figure. Schematic view of the goniometer. Proprioceptive tasks were carried out in a sitting position with a cloak impeding visual feedback. Upper limb position was indicated by a scale at the back of the goniometer. For clarity reasons, the handlebar within the mobile cast is not shown. [file 12883_2020_1858_MOESM1_ESM.docx]

**
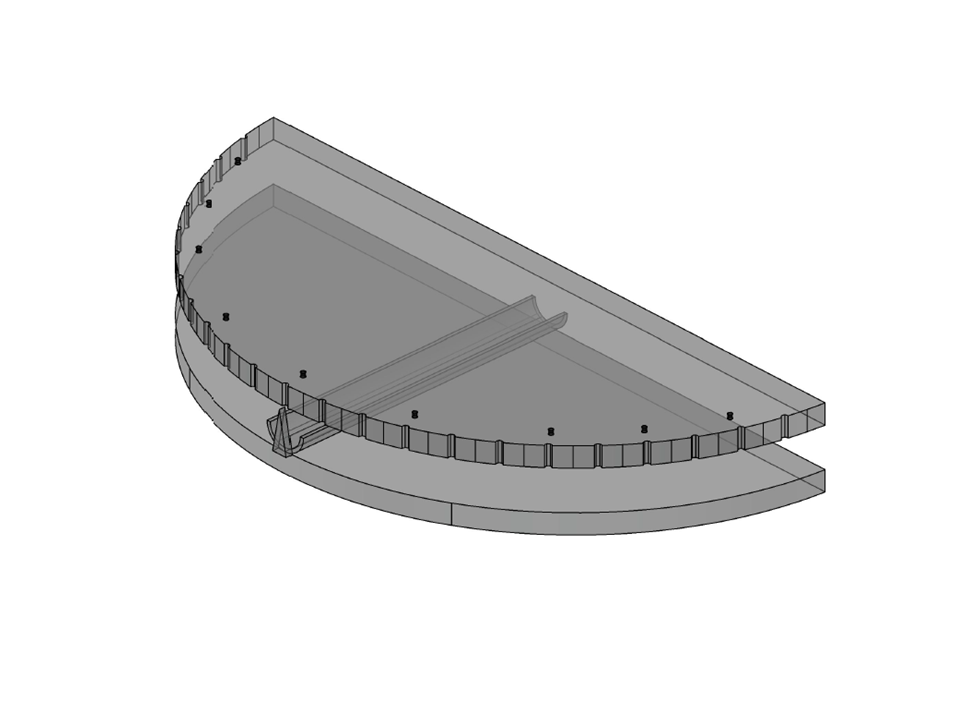
**

**Supplementary Figure 1:** Schematic view of the goniometer. Proprioceptive tasks were carried out in a sitting position with a cloak impeding visual feedback. Upper limb position was indicated by a scale at the back of the goniometer. For clarity reasons, the handlebar within the mobile cast is not shown.
